# Design and descriptive results of the "Growth, Exercise and Nutrition Epidemiological Study In preSchoolers": The GENESIS Study

**DOI:** 10.1186/1471-2458-6-32

**Published:** 2006-02-15

**Authors:** Yannis Manios

**Affiliations:** 1 Department of Nutrition & Dietetics, Harokopio University of Athens, Greece

## Abstract

**Background:**

The Growth, Exercise and Nutrition Epidemiological Study in preSchoolers (GENESIS) attempts to evaluate the food and nutrient intakes, as well as growth and development of a representative sample of Greek toddlers and preschool children. In the current work the study design, data collection procedures and some preliminary data of the GENESIS study are presented.

**Methods:**

From April 2003 to July 2004, 1218 males and 1156 females 1 to 5 years old, stratified by parental educational level (Census 1999), were examined from 105 nurseries in five counties. Approximately 300 demographic, lifestyle, physical activity, dietary, anthropometrical and DNA variables have been recorded from the study population (children and parents).

**Results:**

Regarding anthropometrical indices, boys were found to be taller than girls at all ages (P < 0.05) and heavier only for the age period from 1 to 3 years old (P < 0.05). No significant differences were found between genders regarding the prevalence of at risk of overweight (16.5% to 18.6% for boys and 18.5 to 20.6 % for girls) and overweight (14.0% to 18.9% for boys and 12.6% to 20.0% for girls). Additionally, boys older than 2 years of age were found to have a higher energy intake compared to girls (P < 0.05). A similar tendency was observed regarding the mean dietary intake of fat, saturated fat, carbohydrates and protein with boys exhibiting a higher intake than girls in most age groups (P < 0.05).

**Conclusion:**

The prevalence of overweight in the current preschool population is considerably high. Future but more extensive analyses of the GENESIS data will be able to reveal the interactions of the parameters leading to this phenomenon.

## Background

Infancy and early childhood are both critical periods of rapid physical growth, cognitive and emotional development [1]. Food intake and lifestyle parameters have a central role in this process since they could ensure optimum growth and development on the one hand but also set the basis for a healthy transition through adulthood with lower predisposition for chronic diseases. Several studies have indicated that the roots of chronic disease, both the clinical manifestation of the diseases as well as certain lifestyle patterns and food preferences, may be set in childhood and track into adulthood [2-5].

The realization that the adoption of these health related behaviours may set early in life and is very much determined by internal (i.e family) and external (i.e health professionals, media, food industry) to the child's environment parameters [6], has underlined the need for nutritional surveys in childhood. These surveys are aiming to examine the health and nutritional status of children as well as all the parameters involved in this process, including the health knowledge and practices of parents and caregivers. Expanding our knowledge and understating of the interactions of all these parameters will help and guide public health authorities in developing effective directions both towards the food industry but also towards health promoting bodies, including the family itself.

Several national epidemiological surveys have been conducted so far in industrialized countries, aiming to achieve this goal. Typical examples of such studies are the National Health and Nutrition Examination Surveys (NHANES) I, II and III [7], the Continuing Survey of Food Intakes by Individuals (CSFII) [8] and the 1987–1988 Nationwide Food Consumption Survey (NFCS) [9]. Similar surveys have been conducted in Europe either at a pan European [10] or a country level, using nationally representative samples [11-15] or just small regional cohorts of preschoolers [16-19]. In Greece similar data are only scarce, while probably the most comprehensive data are those provided by Roma-Giannikou et al. [20] on a stratified multiform sample of 2–14 years old children conducted about ten years ago.

The Growth, Exercise and Nutrition Epidemiological Study in preSchoolers (GENESIS) is a large-scale epidemiological study conducted in Greece, attempting to assess growth, development and nutritional status of preschool children. The current paper attempts to provide some information on the study design and data collection procedures, sample's characteristics, as well as some first descriptive results.

## Methods

### Sampling

The study was carried out from April 2003 to July 2004. A representative number of randomly selected public and private nurseries as well as day-care centers within municipalities in five counties (namely Attica, Aitoloakarnania, Thessalonica, Halkidiki and Helia) were invited to participate in the study. All nurseries invited to participate responded positively. Furthermore, an extended letter explaining the aims of the current study and a consent form was provided to each parent or guardian having a child in these nurseries. Those parents agreed to participate in the study had to sign the consent form and provide their contact details. Signed parental consent forms were collected for 2518 children, aged 1 to 5 years old (Response rate 75%). From the total number of positive responses complete data became available for 2374 children with participation rate varying from 54% to 95%, reaching the highest rates in rural areas and the lowest ones in urban areas.

**Figure 1:**
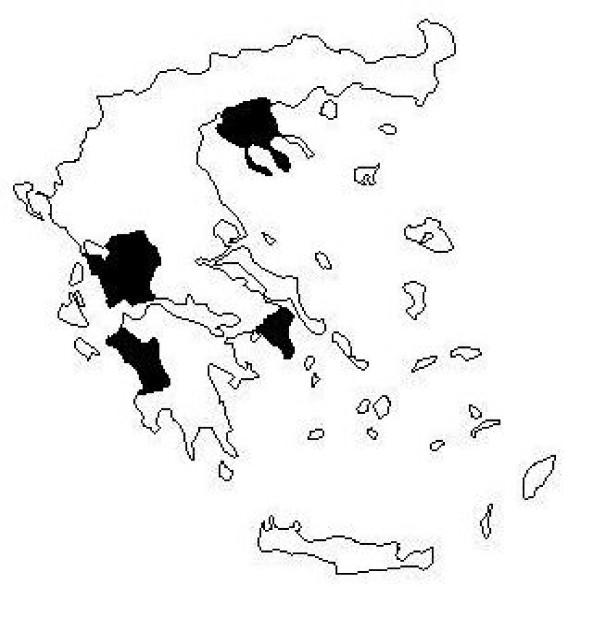
Counties of Greece studied.

These counties are widely scattered over the Greek dominion (Figure 1) while their overall local population comprises about 70% of the total Greek population (Census 1999). Among the total number of nursery schools studied (n = 105), 63 were in Attica, 8 were in Thessalonica, 12 were in Halkidiki, 22 were in Aitoloakarnania and 7 were in Helia. The sampling of the nurseries was random, multistage and stratified by the total population of children, according to data provided by the National Statistical Service of Greece (Census 1999).

The participating regions were grouped based on their population in "Large Urban Areas", with a population size greater than 1,000,000; "Urban Areas" with a population size ranging from 10,000 to 100,000; and "Rural Areas and Small Towns" with a population size less than 10,000 inhabitants. The aforementioned classification stems from a particularity of the population distribution in Greece, according to which there are plenty of "Rural Areas and Small Towns", as well as "Urban" areas but only two "Large Urban" areas of approximately 1,200,000 and 4,500,000 citizens, respectively.

Approval to conduct the study was granted by the Ethical Committee of Harokopio University of Athens and by all municipalities invited to participate in the study.

### Anthropometrical Measurements

All study sites used the same measuring equipment and procedures. The instruments needed to be highly accurate and precise, yet portable enough to be carried to the nursery schools, where the measurements took place. Measurements were taken and recorded by two well-trained team members, which are referred as "leading" and assisting" observer, respectively. The role of the "assisting" observer was to help position the child correctly to the instruments, while the "leading" observer recorded the measurements.

Body weight was recorded to the nearest 10 gr with the use of a Seca digital scale and with subjects standing without shoes in the minimum clothing possible, i.e. underwear. Recumbent length was measured for all subjects to the nearest 0.1 cm with a portable measuring wooden board that had a stationary head piece, a sliding vertical foot piece and a horizontal back piece with a measure tape mounted on it. Further to recumbent length, standing height was also measured to the nearest 0.1 cm in children older than two years of age, with the use of a commercial stadiometer (Leicester Height Measure). The measurement of height was conducted without shoes and with children keeping their shoulders in a relaxed position, their arms hanging freely and with their head aligned in Frankfurt plane. Body Mass Index (BMI) was calculated by dividing weight (Kg) with standing height squared (m^2^).

Head, waist, hip and right arm circumferences were measured to the nearest 0.1 cm with the use of a non-elastic tape (Hoechstmass, Germany) and with the subject at a standing position. More specifically, head circumference was measured to the nearest 0.1 cm after aligning the head in Frankfurt plane and passing the measure tape around the head, just above the eyebrows, above the ears on each side and over the occipital prominence at the back of the head to its maximal circumference. Waist circumference was measured at the end of a gentle expiration after placing the measuring tape in a horizontal plane around the trunk, at the level of umbilicus midway between the lower rib margin and the iliac crest. Hip circumference was measured at the point yielding the maximum circumference over the buttocks. Right arm circumference was measured at the mid-upper-arm point, which is half the distance between the acromion and the olecranon.

The thickness of four skinfolds (triceps, biceps, subscapular and suprailiac) was measured to the nearest 0.1 mm to the right side of the body with a Lange skinfold caliper (Cambridge, Maryland). Each skinfold was grasped gently, in order to avoid causing any unnecessary discomfort to the child. Triceps and biceps skinfold thickness was measured with the right arm hanging relaxed at the side of the body while the skinfold was picked up about 1 cm bellow the midpoint mark over the triceps and biceps muscle respectively. Measurement of the subscapular skinfold thickness was performed while the child stood with shoulders relaxed and after identifying the inferior angle of the scapula. The skinfold was picked up 1 cm below the subscapular mark. Suprailiac skinfold was measured just above the iliac crest, along the axis of the anterior line. In each case the caliper was applied to the "neck" of the fold just above the finger and thumb, for two repeated measurements.

### Dietary Assessment

Dietary intake data was obtained for 3 days (2 consecutive weekdays and 1 weekend day) using a combination of techniques comprising the weighed food records (during nursery hours) and 24 h recall or food diaries (outside nurseries, under parents/guardians supervision). More specifically, during the two weekdays, and while the child was at the nursery, a team member weighed and recorded all foods consumed by each child. Information on the food consumed outside the nursery was obtained during a prearranged meeting with the parent or guardian at the following day. During the interview each parent or guardian was familiarized with portion sizes and the relevant procedures in order to successfully complete a food record at home in the forthcoming weekend day, most preferably Sunday. Parents were advised to return the food diaries at the nursery on Monday morning, where a team member received and checked the records for any miss-recorded or missing information.

Food intake data were analyzed using the Nutritionist V diet analysis software (First Databank, San Bruno, CA), which was extensively amended to include traditional Greek foods and recipes, as described in Food Composition Tables and Composition of Greek Cooked Food and Dishes [21,22]. Furthermore, the databank was updated with nutritional information of chemically analyzed commercial food items widely consumed by the preschool and infant population of Greece. The distribution of usual intakes was estimated by using the National Research Council method (NRC method) [23], which attempts to remove the effects of day-to-day variability (within subject) in dietary intakes. More specifically, the equation used for the calculation of adjusted (usual) intake was the following:

[(Subject's mean - group mean) × SD_between-person_/SD_observed_] + group mean

### Physical activity assessment

Information on children's physical activity was obtained by the parent/guardian during the interview conducted with the assistance of a valid, structured questionnaire [24]. Guardians were asked to provide information of the child's participation in outdoor organized or non-organized physical activities while a research team member was recording this information on this proxy questionnaire. Emphasis was placed on Light to Vigorous Physical Activities (L-VPA) with intensity higher than 4 Metabolic Equivalents (METs). Typical L-VPAs for the younger age groups were playground recreational activities and taking walks with parents while sports participation, such as soccer, swimming, ballet, gymnastics etc were more commonly reported for the older age group. In general L-VPA consisted of all outdoor activities that cause heavy breathing to the child and that have a duration longer than 20 minutes, taking occasional breaks. This approach is not as strict as in adults and older children, which requires 30 minutes of continuous activity, causing heavy breathing and sweating.

### Additional information obtained by parents

During the morning interview at the nursery, additional information were obtained by the guardians concerning (a) demographic characteristics, such as parent's age, employment status and years of education; (b) anthropometrical data, such as current height and body weight of both parents and of child's siblings, as well as a weight history of mother's before conception, before and after child-birth; (c) medical history for siblings, parents and grandparents; (d) smoking and alcohol consumption during pregnancy and breastfeeding, as well as vitamins and/or minerals supplements used during pregnancy; (e) children's feeding patterns from birth to six months of age; (f) parent's perceptions and concerns regarding their children's current eating behaviors, weight status, growth and physical activity levels. Furthermore, parents were asked to bring with them the medical record of their child from where information on child's weight, length and head circumference for the first 12 months of life were copied. Additional information on child's medical history, infections, health problems and intolerances were also recorded.

### DNA sample collection

Buccal cells were collected with a non-invasive and completely painless method, by twirling a sterilized swab on the child's inner cheek for about 10 seconds. The head of the swab was then immersed in a 2 ml microcentrifuge tube, whereas the collected buccal cells would be further isolated for DNA extraction.

### Definition of overweight

The Nutstat module of EpiInfo [25] was used to determine the age and sex-specific percentiles for weight, length and BMI, according to the Centers for Disease Control (CDC) 2000 Growth Charts [26]. Using the CDC weight-for-length growth charts children up to 24 months of age were classified as underweight (≤ 5^th ^percentile) and overweight (≥ 95^th ^percentile). For children older than 24 months, the CDC BMI-for-age growth charts were used for their categorization as underweight (≤ 5^th ^percentile), at risk of overweight (85^th^–95^th ^percentile) and overweight (≥ 95^th ^percentile).

### Statistical Analysis

Descriptive statistics of continuous variables were expressed as Mean ± Standard Deviation (SD) and as proportions for categorical ones. Non-normally distributed variables were logarithmically transformed prior to the analysis. Differences between males and females were evaluated with the unpaired Student's t-test for normally distributed variables and with the non-parametric univariate Mann-Whitney test for skewed variables. Furthermore the chi-square test was used to compare proportions between sexes. The statistical analysis was carried out using the SPSS 11.0 statistical software package for Windows. The level of significance was set at p ≤ 0.05.

## Results

The mean age of all children surveyed was 41.4 ± 11.0 months. The main demographic characteristics of the under study population and the total number of children examined by region of residence is presented in Table 1. Gender was equally distributed in all age groups and regions or residence. Still more subjects were recruited from Large Urban areas reflecting the distribution of the overall population in Greece.

The study population could be considered representative at a county level, since parental distribution by age and educational level was similar to that reported by the National Statistical Service (Census 1999) for the overall population of the respective county (Table 2). Based on this finding and in agreement with the National Statistical Service the prevalence of highly educated parents is higher in "Large Urban Areas" compared to "Urban Areas" and "Rural Areas and Small Towns".

Table 3 shows some anthropometric characteristics of the study sample, by gender and age group. According to these data, male subjects in all age groups were found to be significantly taller (P < 0.05) compared to their female counterparts. Mean weight was found to be higher for boys compared to girls for the first and second age groups (P < 0.001 and P = 0.018 respectively) while no such differences were observed in the other age groups. The prevalence of "overweight" for the first age group was found to be 16.8% and 12.6% for boys and girls respectively (Table 3). For older children the overall prevalence of "at risk of overweight" and "overweight" showed an increasing tendency with age (from 32.6% to 35.4% for boys and from 32.6% to 40.5% for girls). No differences were found between genders regarding mean BMI values and the prevalence of "at risk of overweight" and "overweight" for all age groups.

Mean values of certain behavioural and lifestyle characteristics of the study population are further presented in Table 3. Regarding nutrients intake no significant differences between genders were observed for the first age group. In all other age groups, although total and saturated fat, protein and carbohydrates intake were all higher in boys than girls (P < 0.05), there was no difference between genders in the percentage of energy derived from these macronutrients. Regarding time devoted to L-VPA and TV watching, the current analysis revealed no significant differences between genders as well as that most of the very young children examined didn't devote any time to L-VPA.

## Discussion

In the current paper the study design, data collection procedures and some descriptive data of the GENESIS study are presented. Males in almost all age groups had higher mean values for weight, recumbent length and height, compared to their female counterparts. Furthermore, the mean values obtained for these anthropometrical indices by sex and age group were similar to those reported by national surveys from other developed countries [10,27], indicating similar growth patterns of preschoolers in the developed world. However, and in agreement with other national studies [7,12,14], no differences were detected between genders in BMI values for all age groups, indicating a parallel age-related rise of weight and height in both sexes at preschool years.

The current study is the first to report on the prevalence of overweight on a representative population sample of Greek preschoolers, revealing an increased prevalence of "at risk of overweight" (16.5% to 18.6% for boys and 18.5 to 20.6 % for girls) and "overweight" (14.0% to 18.9% for boys and from 12.6% to 20.0% for girls) (Table 3). The magnitude of these figures can be better assessed when compared with those obtained from a representative US population with similar age (11.0% and 9.9% of boys as well as 9.4% and 11% of girls were found to be at "risk for overweight" and "overweight" respectively) [28,29], indicating a much higher prevalence of "at risk of overweight" and "overweight" among Greek preschoolers. The alarming rate of these finding can be easily realized when considering that currently obesity among adults in the US and Greece are comparable [30], while an increasing rate of obesity among children and adolescents has been recorded in Greece over the last two decades [31,32]. Taking into account recent studies suggesting that childhood obesity in most cases tracks into adulthood [2,4,33,34], the current findings are indicating an increased risk for even higher rates of obesity in adolescence and adulthood in the near future exceeding those currently reported for the US and Greek adult populations.

Overweight and obesity is the outcome of a positive energy balance over a long period of time, i.e. higher energy intake versus lower energy expenditure. Although several recent studies conducted in the developed world have indicated that the increased prevalence of obesity among both children and adults is primarily due to reduced levels of physical activity [35-37] , little is known about preschool years [38]. In the current study both energy intake as well as time spent in physical activity were assessed in order to provide some information on those behavioural parameters related to the increased prevalence of obesity recorded among GENESIS subjects. Mean daily intake of energy and macronutrients was found to be increasing with age for both genders. No differences were observed in these variables between boys and girls at the first age group while from the second age group onwards there was a significantly higher intake for boys compared to girls. Due to the scarcity of relevant epidemiological data among preschool children and toddlers in Greece, no comparisons or conclusive remarks can be reached. Still in the study of Roma-Giannikou et al. [20] similar intakes of energy and macronutrients were reported, indicating that possibly no major changes have taken place in the past ten years. When comparing the current data with those obtained by the NHANES III study [39] for similar age groups, American preschoolers were found to have higher mean energy intake but at the same time lower mean fat and saturated fat intakes compared to the current population. Nevertheless, the percentage of energy derived from total and saturated fat in the GENESIS study (approximately 40.0% and 14.5%, respectively) considerably exceeded those reported for American preschoolers (32.8% and 13.0%, respectively) [39].

In addition to high-energy intake, low levels of physical activity is the other major factor contributing in the development of obesity in both adults and children [35,40]. Still this remains unproven for preschool years [36], probably due to the fact that physical activity may have smaller variations in children compared to adults, thus not providing the significant correlations observed in adults. The present study revealed that most of the children surveyed were not considerably active, since the time spent in L-VPA did not exceed the recommended levels of 30 minutes participation in organized sports and/or free play per day [41]. Furthermore time spent on sedentary activities, such as TV watching and playing video games showed an increasing tendency with age for both genders. These observations are comparable with those reported for preschool children in other developed countries [36], where high rates of childhood obesity co-exist.

The primary aim of the current paper was to present the study design and data collection procedures and also to provide some preliminary crude/ descriptive findings of the GENESIS study. The data presented have indicated an increasing tendency with age regarding the overall prevalence of at risk of overweight and overweight. Future but more extensive analyses of the GENESIS data will be able to provide more evidence on the behavioural parameters related to these increased rates. Most importantly however, a more thorough analysis of the available data will try to highlight the cultural, parental and socio-economic indices, leading to the unfavourable behavioural parameters related to this phenomenon. Expanding the knowledge and understanding of the interactivity of these parameters will assist Public Health Bodies in developing appropriate health and nutrition guidelines for Health Educators and Health Professionals but also provide guidance for the food industry, parents and guardians. This approach could lead to maximizing the efficiency of health promotion strategies, both towards the direction of preventing chronic disease but also achieving optimum growth and development for preschool children.

## Pre-publication history

The pre-publication history for this paper can be accessed here:



## Figures and Tables

**Table 1:** Description of the study population by gender, age group and region of residence.

	**Boys**		**Girls**	
		
	n	%	n	%
**Age Group**				
First (1–2 yrs old)	100	8.2	107	9.3
Second (2–3 yrs old)	274	22.5	226	19.6
Third (3–4 yrs old)	488	40.1	434	37.5
Fourth (4–5 yrs old)	356	29.2	389	33.7
**Region of Residence**				
Rural Areas & Small Towns	261	21.4	247	21.4
Urban Areas	281	23.1	252	21.8
Large Urban Areas	676	55.5	657	56.8
**Total**	1218		1156	

**Table 2:** Parental educational level by county

	Counties									
	
	Attica* (n = 1257)		**Etoloakarnania^†^** **(n = 449)**		**Halkidiki^†^** **(n = 319)**		**Helia^†^** **(n = 155)**		Thessalonica* (n = 194)	
	n	(%)	n	(%)	n	(%)	n	(%)	n	(%)
**Father's educational level**										
Illiterate- elementary	35	(2.7)	43	(9.6)	32	(10.1)	7	(4.4)	5	(2.5)
Junior high school	57	(4.5)	62	(13.7)	60	(18.7)	31	(20.3)	25	(13.1)
High school	450	(35.8)	186	(41.5)	114	(35.9)	59	(38.0)	62	(31.7)
Further education	715	(56.9)	158	(35.2)	113	(35.3)	55	(37.3)	102	(52.8)
**Mother's educational level**										
Illiterate- elementary	19	(1.5)	29	(6.5)	7	(2.1)	7	(4.5)	9	(4.5)
Junior high school	50	(4.0)	44	(9.8)	40	(12.6)	14	(8.9)	16	(8.5)
High school	370	(29.4)	178	(39.7)	116	(36.5)	53	(34.4)	48	(24.5)
Further education	818	(65.1)	198	(44.0)	156	(48.8)	81	(52.2)	121	(62.5)

The sampling was random, multistage and stratified by parental educational level according to data provided by the National Statistical Service of Greece (Census of 1999).

*: "Large Urban Areas"

^†^: "Urban Areas" and "Rural Areas and Small Towns"

**Table 3:** Descriptive statistics of important anthropometric, dietary and behavioral variables tested, by age group.

**Variables**	**First Age Group (12–24 months old)**				
	**Boys**		**Girls**		**P-value**
	
**Anthropometrical**	**Mean ± s.d**		**Mean ± s.d**		
Weight^§^(Kg)	12.7 ± 1.8		11.7 ± 1.8		<0.001
Height^† ^(cm)	85.4 ± 5.8		82.6 ± 4.8		<0.001
BMI (Kg/m^2^)	-		-		-
	**n**	**%**	**n**	**%**	
Underweight	1	1.0	2	2.1	0.573
At risk of overweight	**-**	**-**	**-**	**-**	-
Overweight	16	16.8	12	12.6	0.380
**Nutrient Intake**	**Mean ± s.d**		**Mean ± s.d**		
Energy^§ ^(MJ/day)	5.35 ± 0.88		5.22 ± 0.75		0.312
(Kcal/day)	1277.4 ± 211.3		1246.9 ± 178.5		
Total Fat^§ ^(gr/day)	53.0 ± 13.4		51.4 ± 11.6		0.394
Total Fat (% of Energy)	37.1 ± 5.2		36.9 ± 5.3		0.772
Saturated Fat^§ ^(gr/day)	19.9 ± 7.4		19.3 ± 5.2		0.672
Saturated Fat^§ ^(% of Energy)	13.8 ± 3.3		13.8 ± 2.7		0.910
Carbohydrates^§ ^(gr/day)	154.0 ± 26.5		151.2 ± 24.9		0.472
Carbohydrates^§ ^(% of Energy)	48.4 ± 5.3		48.6 ± 5.5		0.792
Protein^§ ^(gr/ day)	52.2 ± 10.7		50.5 ± 9.6		0.287
Protein^§ ^(% of Energy)	16.3 ± 1.8		16.2 ± 1.7		0.597
**Behavioral**	**Mean ± s.d**		**Mean ± s.d**		
TV watching (hrs/day)*	0.55 ± 0.88		0.46 ± 0.80		0.671
L-VPA (hrs/week)*	1.45 ± 3.15		1.05 ± 2.29		0.498

**Variables**	**Second Age Group (25–36 months old)**				

	**Boys**		**Girls**		**P-value**
	
**Anthropometrical**	**Mean ± s.d**		**Mean ± s.d**		
Weight^§^(Kg)	15.2 ± 2.5		14.7 ± 2.2		0.018
Height^† ^(cm)	95.4 ± 4.5		94.1 ± 4.5		0.008
BMI (Kg/m^2^)	17.2 ± 2.4		17.0 ± 2.0		0.208
	**n**	**%**	**n**	**%**	
Underweight	9	3.5	7	3.3	0.919
At risk of overweight	48	18.6	39	18.5	0.973
Overweight	36	14.0	32	15.2	0.711
**Nutrient Intake**	Mean ± s.d		Mean ± s.d		
Energy^§ ^(MJ/day)	5.84 ± 0.95		5.61 ± 0.91		0.007
(Kcal/day)	1394.9 ± 227.5		1338.0 ± 218.6		
Total Fat^§ ^(gr/day)	62.5 ± 13.6		59.3 ± 13.8		0.008
Total Fat (% of Energy)	40.2 ± 4.4		39.7 ± 4.9		0.238
Saturated Fat^§ ^(gr/day)	23.1 ± 6.3		21.6 ± 5.9		0.004
Saturated Fat^§ ^(% of Energy)	14.8 ± 2.7		14.4 ± 2.7		0.075
Carbohydrates^§ ^(gr/day)	157.5 ± 29.6		152.5 ± 27.2		0.085
Carbohydrates^§ ^(% of Energy)	45.2 ± 5.0		45.8 ± 5.2		0.264
Protein^§ ^(gr/ day)	57.8 ± 11.7		55.2 ± 12.6		0.013
Protein^§ ^(% of Energy)	16.6 ± 2.1		16.5 ± 2.3		0.465
**Behavioral**	Mean ± s.d		Mean ± s.d		
TV watching (hrs/day)*	1.20 ± 1.20		1.18 ± 1.23		0.527
L-VPA (hrs/week)*	1.51 ± 2.63		1.21 ± 2.41		0.377

**Variables**	**Third Age Group (37–48 months old)**				

	**Boys**		**Girls**		**P-value**
	
**Anthropometrical**	**Mean ± s.d**		**Mean ± s.d**		
Weight^§^(Kg)	17.3 ± 2.5		17.0 ± 2.7		0.098
Height^† ^(cm)	103.0 ± 4.7		102.1 ± 4.6		0.003
BMI (Kg/m^2^)	16.8 ± 1.8		16.6 ± 1.8		0.307
	**n**	**%**	**n**	%	
Underweight	15	3.2	12	2.9	0.808
At risk of overweight	79	17.0	84	20.6	0.173
Overweight	90	19.4	62	15.2	0.106
**Nutrient Intake**	**Mean ± s.d**		**Mean ± s.d**		
Energy^§ ^(MJ/day)	6.04 ± 0.99		5.78 ± 0.99		<0.001
(Kcal/day)	1442.4 ± 237.1		1379.0 ± 237.3		
Total Fat^§ ^(gr/day)	65.1 ± 14.3		62.3 ± 14.2		0.004
Total Fat (% of Energy)	40.4 ± 4.5		40.5 ± 4.6		0.857
Saturated Fat^§ ^(gr/day)	23.5 ± 6.3		22.5 ± 6.3		0.024
Saturated Fat^§ ^(% of Energy)	14.6 ± 2.8		14.6 ± 2.8		0.779
Carbohydrates^§ ^(gr/day)	161.9 ± 30.1		154.6 ± 29.2		<0.001
Carbohydrates^§ ^(% of Energy)	45.0 ± 4.8		45.0 ± 4.8		0.991
Protein^§ ^(gr/ day)	59.8 ± 12.7		56.9 ± 12.6		<0.001
Protein^§ ^(% of Energy)	16.6 ± 2.2		16.5 ± 2.1		0.505
**Behavioral**	Mean ± s.d		Mean ± s.d		
TV watching (hrs/day)*	1.89 ± 1.53		1.81 ± 1.32		0.780
L-VPA (hrs/week)*	1.27 ± 2.52		1.09 ± 2.09		0.851

**Variables**	**Fourth Age Group (49–60 months old)**				

	**Boys**		**Girls**		**P-value**
	
Anthropometrical	**Mean ± s.d**		**Mean ± s.d**		
Weight^§^(Kg)	19.6 ± 3.2		19.2 ± 3.4		0.116
Height^† ^(cm)	109.8 ± 4.8		108.8 ± 5.3		0.006
BMI (Kg/m^2^)	16.6 ± 1.9		16.6 ± 2.0		0.889
	**n**	**%**	**n**	**%**	
Underweight	11	3.3	8	2.2	0.371
At risk of overweight	55	16.5	75	20.5	0.166
Overweight	63	18.9	73	20.0	0.704
**Nutrient Intake**	**Mean ± s.d**		**Mean ± s.d**		
Energy^§ ^(MJ/day)	6.18 ± 0.12		5.92 ± 0.11		0.009
(Kcal/day)	1474.9 ± 295.8		1414.1 ± 259.7		
Total Fat^§ ^(gr/day)	66.3 ± 17.1		63.9 ± 15.5		0.081
Total Fat (% of Energy)	40.2 ± 5.0		40.5 ± 5.0		0.593
Saturated Fat^§ ^(gr/day)	23.8 ± 7.2		22.7 ± 6.5		0.070
Saturated Fat^§ ^(% of Energy)	14.5 ± 2.9		14.4 ± 2.9		0.948
Carbohydrates^§ ^(gr/day)	167.6 ± 44.3		158.9 ± 39.2		0.014
Carbohydrates^§ ^(% of Energy)	45.2 ± 6.4		44.8 ± 6.5		0.344
Protein^§ ^(gr/ day)	60.5 ± 15.2		57.8 ± 13.7		0.028
Protein^§ ^(% of Energy)	16.4 ± 2.5		16.3 ± 2.3		0.784
**Behavioral**	**Mean ± s.d**		**Mean ± s.d**		
TV watching (hrs/day)*	2.31 ± 1.55		2.16 ± 1.45		0.167
L-VPA (hrs/week)*	1.10 ± 2.42		1.17 ± 2.13		0.092

^†^: The variable "Height" presented in the Table corresponds to recumbent length for children 1 to 2 years old and to standing height for children older than 2 years.

^§^: Parameter was log-transformed

*: The level of statistical significance was estimated with the non-parametric Mann-Whitney test
